# Supplementary material for: Reentrant melting of scarred odd crystals by self-shear
Source: Nat Commun. 2026 Jan 16;17:1802. doi: 10.1038/s41467-026-68510-4 (PMC12916785; doi:10.1038/s41467-026-68510-4)
Supplement: Supplementary file 1 — Supplementary Information [file 41467_2026_68510_MOESM1_ESM.pdf]

Supplementary Material for
"Reentrant melting of scarred odd crystals by self-shear"

Uttam Tiwari,[1] Pragya Arora,[1] A K Sood,[2,3] Sriram Ramaswamy,[2,4] Rituparno Mandal,[5] and Rajesh Ganapathy[1,3,6]

[1] *Chemistry and Physics of Materials Unit, Jawaharlal Nehru Centre for Advanced Scientific Research, Jakkur, Bangalore - 560064, INDIA*
[2] *Department of Physics, Indian Institute of Science, Bangalore - 560012, INDIA*
[3] *International Centre for Materials Science, Jawaharlal Nehru Centre for Advanced Scientific Research, Jakkur, Bangalore - 560064, INDIA*
[4] *International Centre for Theoretical Sciences, Bangalore - 560089, India*
[5] *Soft Condensed Matter Group, Raman Research Institute, Bangalore - 560080, INDIA*
[6] *School of Advanced Materials (SAMat), Jawaharlal Nehru Centre for Advanced Scientific Research, Jakkur, Bangalore - 560064, INDIA*

(Dated: December 28, 2025)

CONTENTS

1. Quantifying the homogeneity of the spinner packings ($\chi = 0$) 2

2. Pair correlation function 3

3. Hexagonal bond-order parameter and radial density profiles 4
   3.1. $|\psi_6^i|$ and radial density at $\phi = 0.68$ 4
   3.2. $|\psi_6^i|$ and radial density at $\phi = 0.75$ 5
   3.3. $|\psi_6^i|$ and radial density at $\phi = 0.79$ 6

4. Microscopic mechanism leading to edge currents 7

5. Quantifying edge and bulk flows 8

6. Self-shearing in simulations - Transient angular velocity profile 8

7. Quantifying finite-size effects on grain-boundary scar configuration, edge flow, and bulk flow 9

8. Phase diagram in (χ, ϕ) plane 11

9. Grain boundary scars in simulations of passive disks for different system sizes 11

10. Grain boundary scars in simulations of spinners 12

11. Radial defect density 13

12. Radial hexagonal bond-order parameter 14

13. Odd moduli in spinner materials 14

14. Grain boundary scars and sector density 16

15. Simulations - Resistive torque profile 16

16. Quantifying spin speeds of spinners 18

17. Radial profile for spin velocity of spinner 18

18. Relating fluctuations in radial spin and angular velocities to the structure of the spinner packing 19

19. Experiments - Vortical flows and radial density profiles 20
    19.1. Vorticity and radial density at $\phi = 0.79$ 20
    19.2. Vorticity and radial density at $\phi = 0.75$ 21
    19.3. Vorticity and radial density at $\phi = 0.68$ 23

20. Supplementary videos 23

References 24

1. QUANTIFYING THE HOMOGENEITY OF THE SPINNER PACKINGS ($\chi = 0$)

To test whether the racemic mixture exhibited a tendency to phase-separate, we analyzed the local distribution of clockwise and counterclockwise spinners over the entire system at $\chi = 0$, $\phi = 0.72$. Specifically, we measured the number of clockwise-spinning nearest-neighbors surrounding a clockwise spinner, and likewise for counterclockwise spinners, at both the beginning and end of the experiment (Supplementary Fig. 1). The resulting distribution is sharply peaked at three and remains unchanged over time. Given that the local coordination number in our experiments is six, this result indicates that each particle is, on average, surrounded by equal numbers of clockwise and counterclockwise spinners.

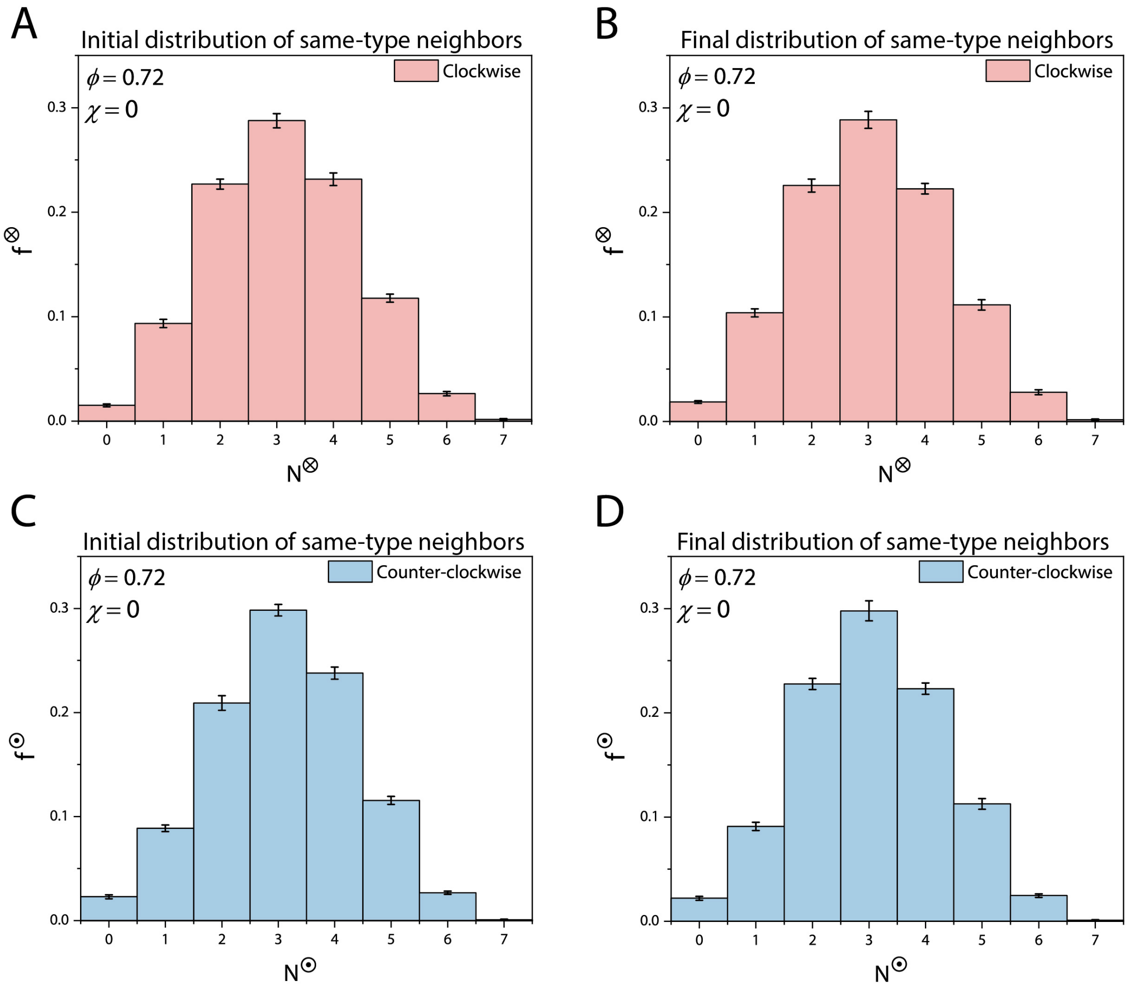

Supplementary Fig. 1: Clockwise and counter-clockwise spinners are uniformly mixed at $\chi = 0$. (**A and B**) Fraction of $\otimes$ spinners with same-type nearest neighbor at $\chi = 0$ and $\phi = 0.72$. (**A**) Distribution at the beginning of the experiment (first 50 seconds). (**B**) Distribution at the end of the experiment (last 50 seconds). (**C and D**) Fraction of $\odot$ spinners with same-type nearest neighbor at $\chi = 0$ and $\phi = 0.72$. (**C**) Distribution at the beginning of the experiment (first 50 seconds). (**D**) Distribution at the end of the experiment (last 50 seconds). The error bars represent the standard error.

2. PAIR CORRELATION FUNCTION

The pair correlation function, $g(r)$, gives information about the local structure of a system [1]. In 3D, it is defined as:

$$g(r) = \frac{V}{4\pi r^2 N^2} \sum_{i=1}^{N} \sum_{j \neq i}^{N} \delta(r - |r_i - r_j|) \tag{1}$$

where N is the total number of particles, and V is the volume. In 2D, the equation becomes:

$$g(r) = \frac{A}{2\pi r N^2} \sum_{i=1}^{N} \sum_{j \neq i}^{N} \delta(r - |r_i - r_j|) \tag{2}$$

where A is the area. We plot $g(r)$ for χ ranging from 0 to 1 at $\phi = 0.68$, $\phi = 0.72$, $\phi = 0.75$, $\phi = 0.79$, respectively (Supplementary Fig. 2). A reentrant in peak heights of $g(r)$ was observed when χ is increased at a fixed ϕ. No reentrant was observed for $\phi = 0.75$ and $\phi = 0.79$. We observed that the peak height is the shortest, consistent with the highest number of defects at $\chi = 0$ for $\phi = 0.75$ and $\phi = 0.79$.

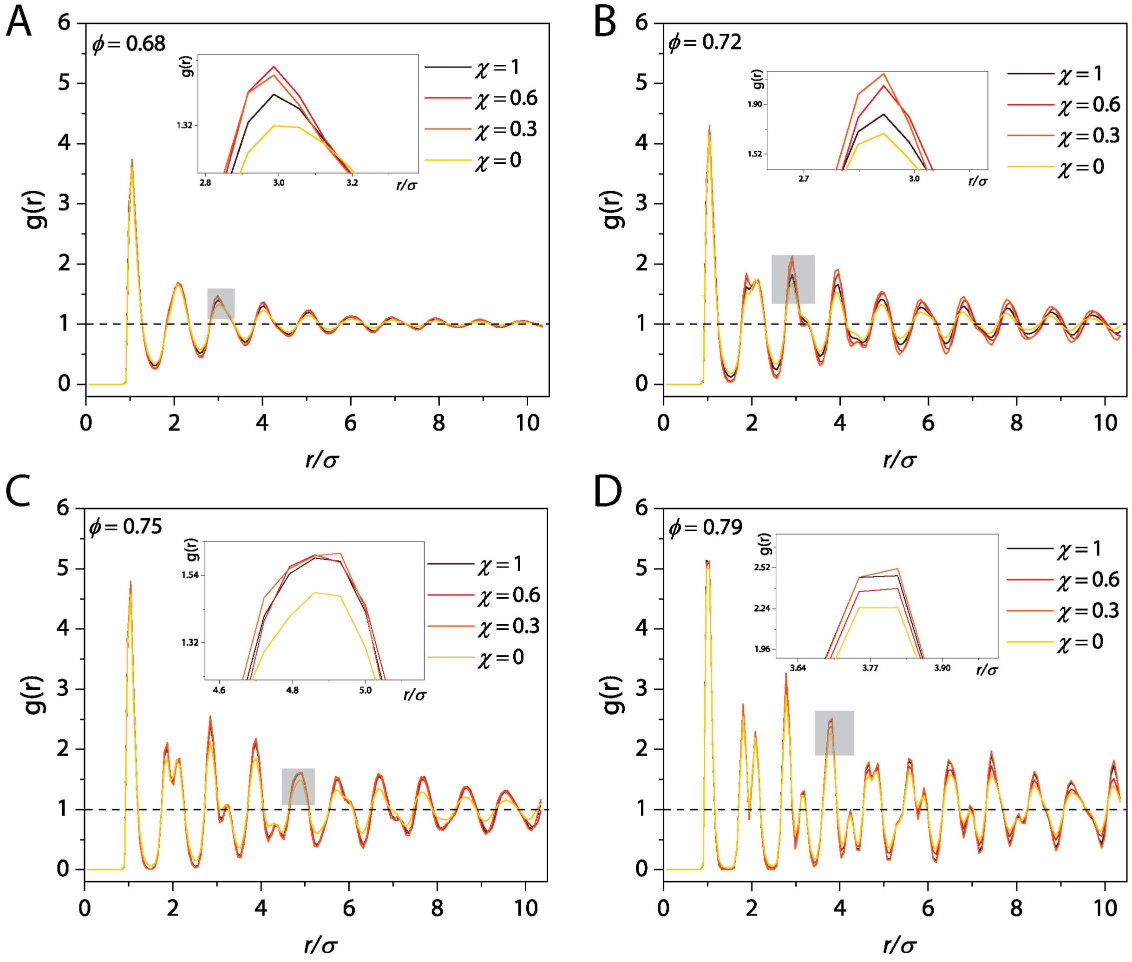

Supplementary Fig. 2: Reentrant in peak-height of the pair correlation function. (**A to D**) Pair correlation function, $g(r)$, for various values of χ for (**A**) $\phi = 0.68$, (**B**) $\phi = 0.72$, (**C**) $\phi = 0.75$, and (**D**) $\phi = 0.79$. (**A to D**) The inset shows the zoomed-in view of the peak heights corresponding to the gray-shaded region. Here σ is the diameter of the spinner.

3. HEXAGONAL BOND-ORDER PARAMETER AND RADIAL DENSITY PROFILES

The Hexagonal bond-order parameter, ψ_6^i, gives information about the arrangement of spinners in the system. Here, $\psi_6^i = \frac{1}{N_i}\sum_{j=1}^{N_i} e^{6i\theta_{ij}(t)}$ where, N_i is the coordination of spinner i and θ_{ij} is the angle between the line joining the center of spinner i to its nearest neighbour j with respect to a fixed axis. To quantify the hexagonal packing, we compute the magnitude of the hexagonal bond-order parameter, $|\psi_6^i|$, of each spinner. The $|\psi_6^i|$ value is 1 for a perfect hexagonal lattice and decreases as the structure deviates from the hexagonal symmetry. We expand on this in detail for $\phi = 0.68$, $\phi = 0.75$, and $\phi = 0.79$ in the following sections.

Since our material is odd, the presence of finite odd material moduli that couple flows to the transverse pressure should directly affect the radial density of spinners in circular confinement. This effect is captured by plotting the radial density, $\phi_A(r)$, as a function of r/R. To obtain $\phi_A(r)$, we divide the system into concentric annuli and plot the number density associated with each annulus as a function of r/R. Here, r is the outer radius of the annulus. The width of the annulus is $0.09d_p$, where d_p is the diameter of the spinner. In the following, we plot $\phi_A(r)$ for $\phi = 0.68$, $\phi = 0.75$, and $\phi = 0.79$ at all values of χ along with $|\psi_6^i|$ for each spinner.

3.1. $|\psi_6^i|$ and radial density at $\phi = 0.68$

Supplementary Fig. 3A shows the arrangement of spinners in circular confinement for χ ranging from 0 to 1 at $\phi = 0.68$. Each spinner is colored based on the $|\psi_6^i|$ value. We observed reentrant behavior at $\phi = 0.68$ similar to that at $\phi = 0.72$ (see Fig. 1C of the Main manuscript). However, the reentrant effect is diminished at $\phi = 0.68$. The radial density, $\phi_A(r)$, shows evident changes as a function of χ (Supplementary Fig. 3B). The large amplitude oscillations near $r/R = 1$ result from spinner layering near confinement. The radial density profile shows enhanced peak height near $r/R = 1$ for $\chi > 0$, indicating the presence of extra radial stress.

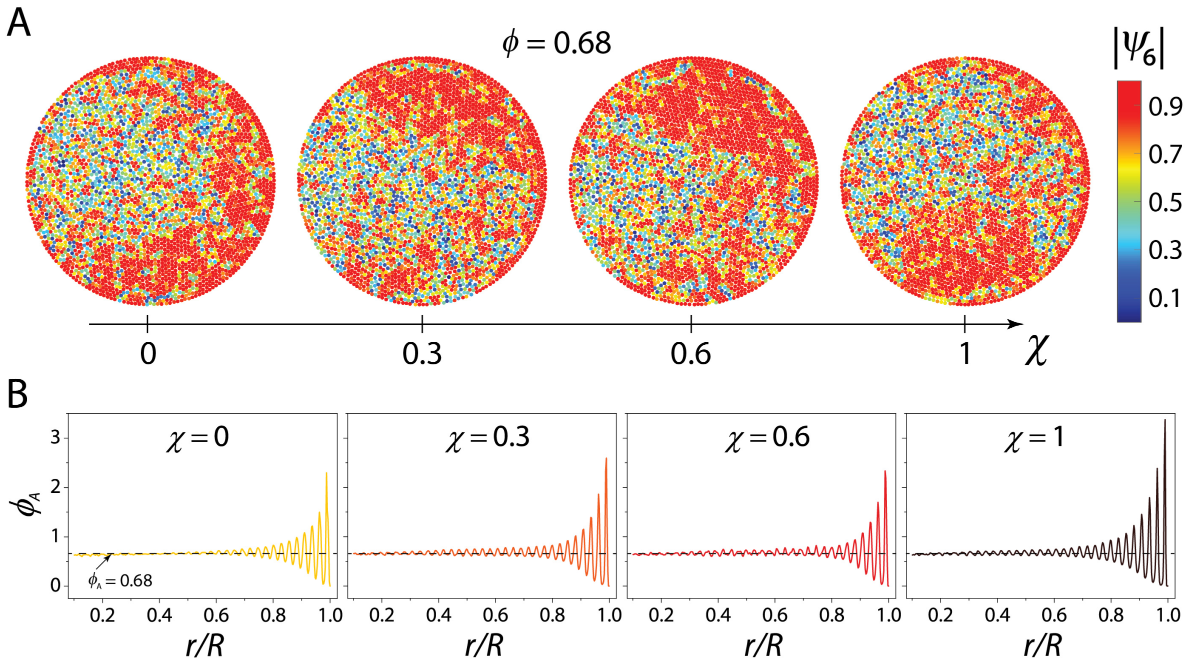

Supplementary Fig. 3: χ driven reentrant melting transition at $\phi = 0.68$. (**A**) The panels show the arrangement of spinners in the assembly at $\phi = 0.68$ for different χ values. Each spinner is colored based on the magnitude of the hexagonal bond-order parameter, $|\psi_6^i|$, in the color bar. (**B**) The radial density, $\phi_A(r)$, as a function of r/R at various χ for $\phi = 0.68$. The dashed line highlights the average density.

3.2. $|\psi_6^i|$ and radial density at $\phi = 0.75$

At $\phi = 0.75$, we observed a small liquid-like pocket near the center for $\chi = 0$, which vanished for $\chi > 0$ (Supplementary Fig. 4A). The peak heights in the radial density profiles near $r/R = 1$ are lower for $\chi = 0$ compared to $\chi > 0$ (Supplementary Fig. 4B). The modification in $|\psi_6^i|$ and $\phi_A(r)$ as a function of χ suggests the presence of excess radial stress for $\chi > 0$. The reentrant behavior observed at $\phi = 0.68$ and $\phi = 0.72$ was notably absent at $\phi = 0.75$, suggesting that the reentrant is sensitive to the compressibility of the system.

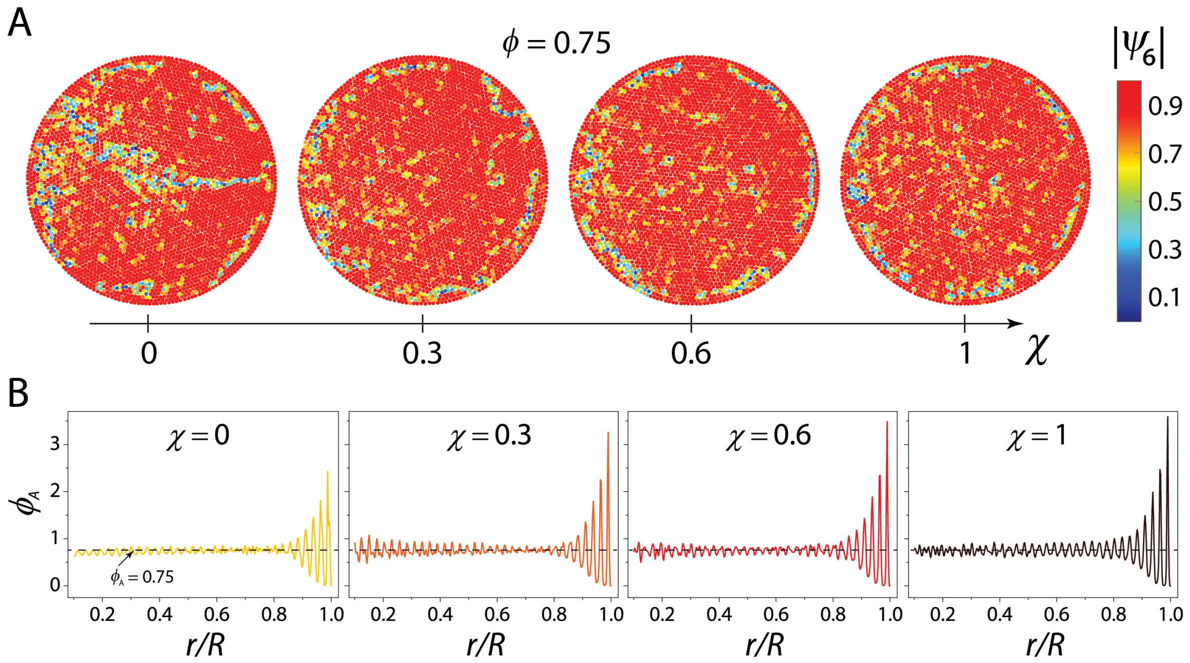

Supplementary Fig. 4: Reentrant melting vanishes when the system becomes less compressible. (**A**) Arrangement of spinners in the assembly at $\phi = 0.75$ for different χ values. Each spinner is color-coded based on the value of $|\psi_6^i|$ in the color bar. (**B**) The radial density, $\phi_A(r)$, as a function of r/R at $\phi = 0.75$ for different value of χ. The dashed line shows the mean density.

3.3. $|\psi_6^i|$ and radial density at $\phi = 0.79$

Unlike other values of ϕ, where we observed a liquid-like pocket in bulk for $\chi = 0$, here, the defects are line-like, corresponding to the grain boundary (Supplementary Fig. 5A). With the increase in χ, we observed an enhancement in the crystallinity. We observed significant changes in the radial density profiles as a function of χ (Supplementary Fig. 5B).

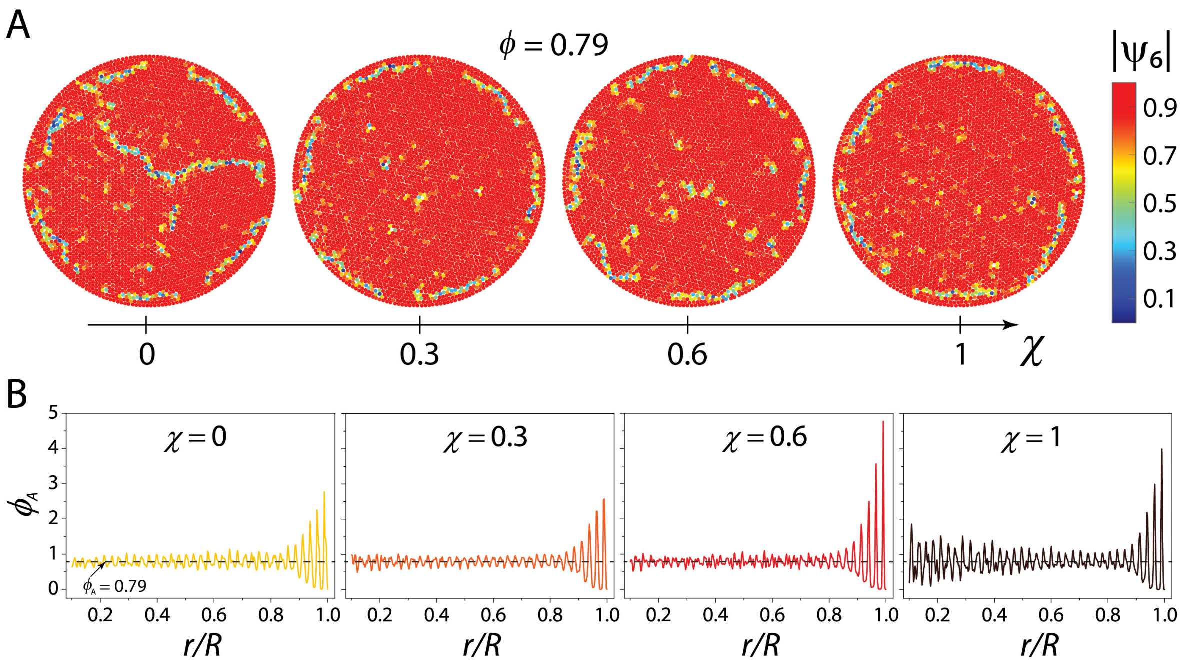

Supplementary Fig. 5: Crystallinity enhances with an increase in χ for an incompressible system. (**A**) Spatial arrangement of spinners at $\phi = 0.79$ for different χ values. Each spinner is color-coded according to the magnitude of the hexagonal bond-order parameter, $|\psi_6^i|$, in the color bar. (**B**) The radial density, $\phi_A(r)$, as a function of r/R at various χ for $\phi = 0.79$. The horizontal dashed line shows the average density.

4. MICROSCOPIC MECHANISM LEADING TO EDGE CURRENTS

Supplementary Fig. 6 shows the arrangement of (⊗) spinners near the confining boundary for $\chi = 1$. As a result of friction, each spinner applies a tangential force on its neighbors. The spinner 2 exerts a tangential force on the spinner 1, F_{12}, in the clockwise direction. The confining boundary exerts a force, F_{10}, on spinner 1 in the anticlockwise direction. If the tangential force, F_{10}, is greater than F_{12}, the emergent edge flow has handedness opposite to the spin of the individual spinner. However, when F_{12} is greater than F_{10}, the edge flow will have the same handedness as the spin of the spinner. For our system, the edge flow and the handedness of spinners are aligned, implying that the frictional interaction between a spinner and the confining boundary is weaker than the friction between two spinners.

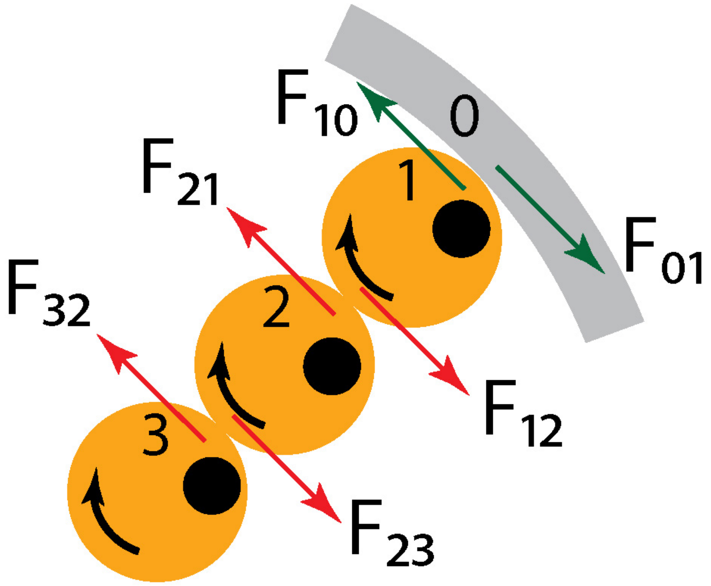

Supplementary Fig. 6: The schematic diagram for tangential forces arising from friction between spinners. The Gray shaded region represents the confining boundary. Red arrows show the tangential forces between spinners, while green arrows show the tangential forces between spinners and the confining boundary.

5. QUANTIFYING EDGE AND BULK FLOWS

Topologically protected edge flow is a hallmark of chiral active systems [2–4]. To quantify these flows in our system, we looked at the annular angular velocity, $\langle\omega(r)\rangle$, as a function of distance from the center. To get $\langle\omega(r)\rangle$ as a function of r/R (Supplementary Fig. 7), we divided the system into concentric annuli such that the width of each annulus is comparable in size to the diameter of a spinner. Here, R is the radius of the confining boundary, and r is the outer radius of the annulus. At $\phi = 0.68$, we observed an edge flow in the same direction as the individual spin of the dominant spinners ($\otimes$) for values of $\chi > 0$. To our surprise, we also observed a sign reversal in $\langle\omega(r)\rangle$ at $r/R \approx 0.9$, which indicates a counter-propagating bulk current. These counter-propagating edge and bulk currents are also present at $\phi = 0.72$ for $\chi = 0.6$ and $\chi = 1$. For $\phi = 0.75$ and $\phi = 0.79$, we did not observe any sign reversal in $\langle\omega(r)\rangle$. In fact, for $\phi = 0.79$, we observed a solid body-like rotation for all values of $\chi > 0$.

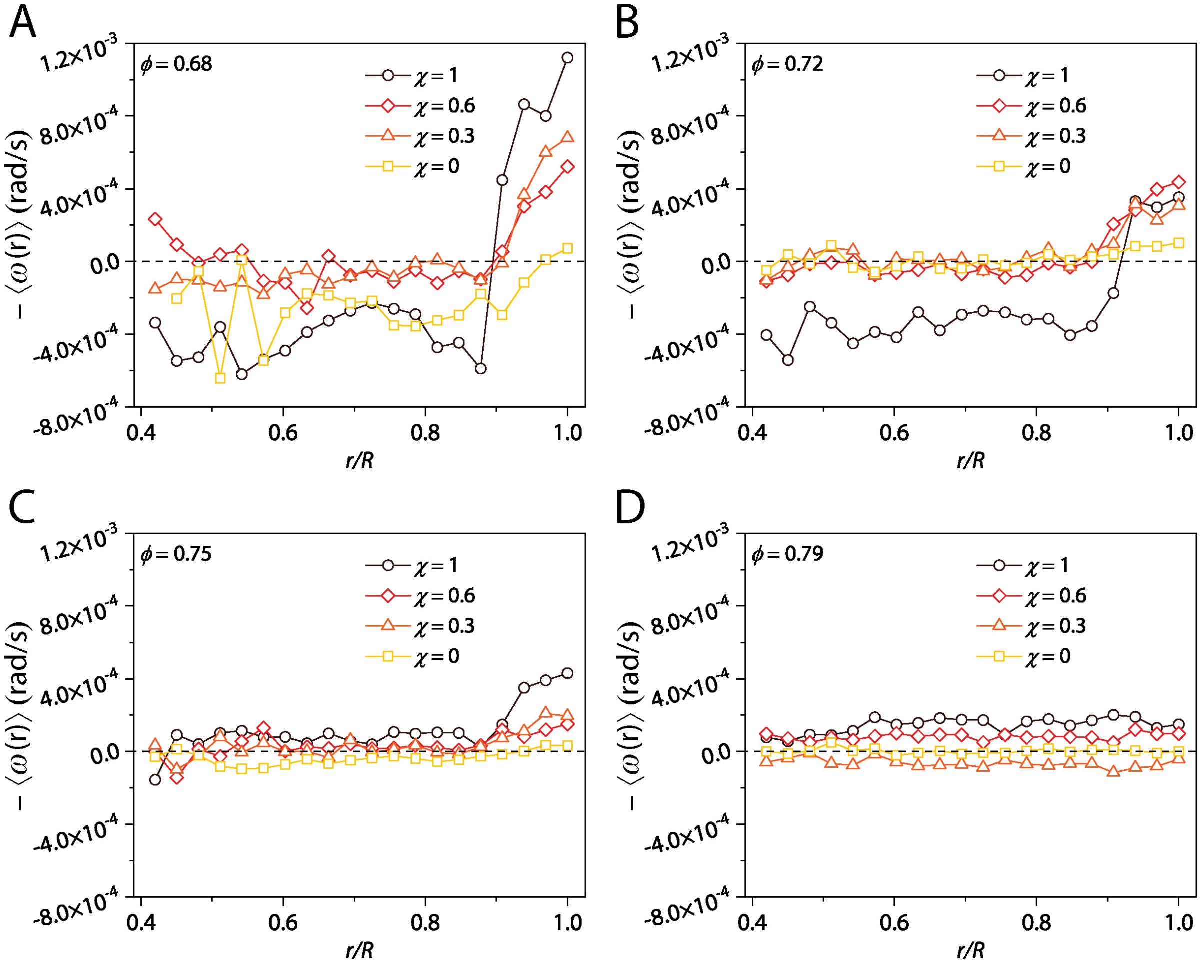

Supplementary Fig. 7: Quantifying the emergent edge and bulk flow. (**A to D**) The average angular velocity of spinners in concentric annuli with respect to the system center, $\langle\omega(r)\rangle$, versus r/R for various values of χ for (**A**) $\phi = 0.68$, (**B**) $\phi = 0.72$, (**C**) $\phi = 0.75$, and (**D**) $\phi = 0.79$. Here, $\langle\rangle$ denotes an average over different spinners in an annulus.

6. SELF-SHEARING IN SIMULATIONS - TRANSIENT ANGULAR VELOCITY PROFILE

In the main text, we described a self-shearing dynamical state where one can observe a counter-rotating flow between the bulk and the boundary layers of the assembly of active granular spinners. We employed our simulation model at different densities ($\phi = 0.68, 0.72, 0.75$ and 0.79) to see whether the simulation also demonstrates a similar

phenomenology. For this particular experiment, we used $N = 3689$ and varying spinner radius R_0, and we explored the effect of different area fractions. We first calculated the transverse (or azimuthal) component of the net velocity for each spinner and computed the contribution for each annulus as $v_T(r)$. Finally, we calculated $\omega(r) = v_T(r)/r$ where r is the radius of the annuli with a transverse velocity $v_T(r)$. For most of the densities $(0.68, 0.72$ and $0.75)$ $\omega(r)$ changes sign near the location of the scar (gray-shaded region in Supplementary Fig. 8), signifying the counter-rotating flows of bulk and boundary. For longer simulations, we see that boundary flow wins and drags the bulk along with it. This is also the case for $\phi = 0.79$ even for a short time scale. While the emergent flows in our experiments are observed in the steady state, in our minimal simulation, we observe such flows only for a transient period. This limitation of our model can potentially stem from the lack of realistic details such as spinning mechanisms (in our simulations, the spinning speed is maintained at a fixed value, almost forcefully), boundary conditions (the boundary is created by a placing set of small particles along a circular perimeter, rather than a continuous circular object), and the modeling of friction (in our simulations friction is modeled by Coulomb friction). Moreover, in the simulations, we use a 2D system that does not capture the complex quasi-2D dynamics of the experiments. Nonetheless, our simulations can reliably capture the counter-rotating nature of the flows, the scar statistics, and the decoupling of bulk-boundary.

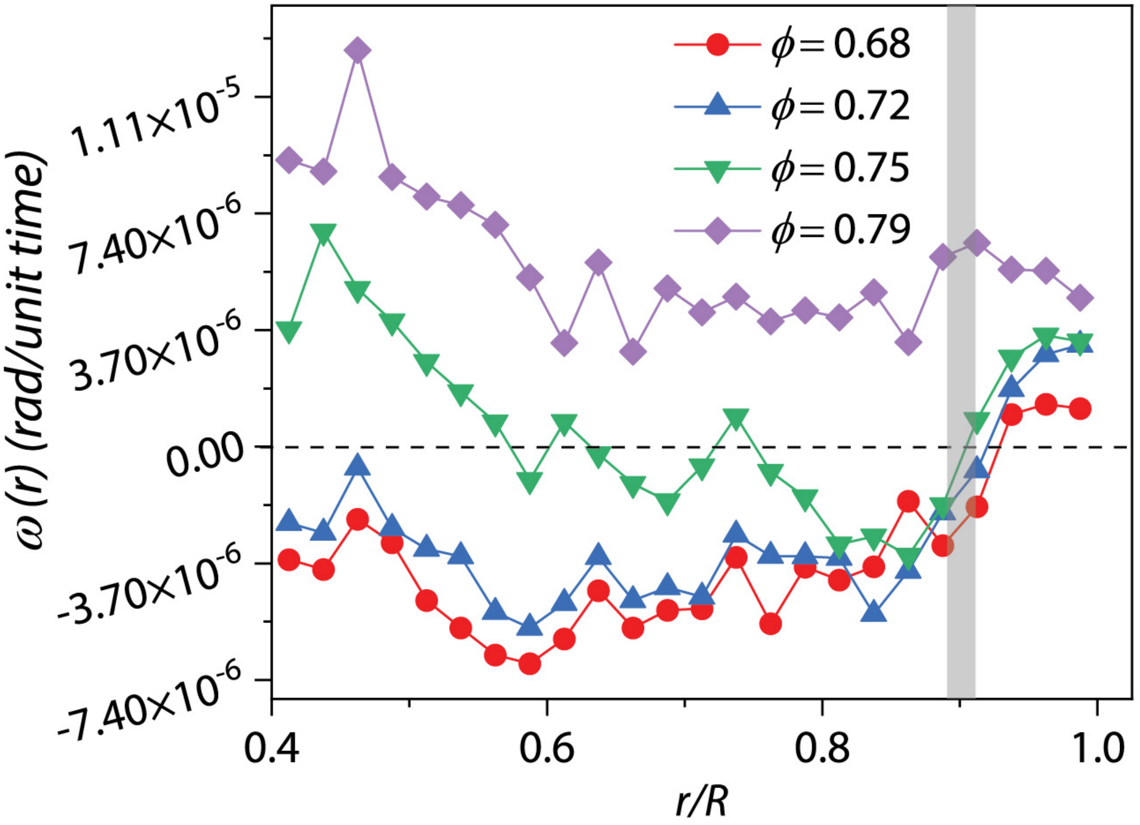

Supplementary Fig. 8: Edge and bulk currents in simulations. Angular speed $\omega(r)$ calculated as a function of radial distance r/R for different area fractions $\phi = 0.68, 0.72, 0.75$ and 0.79. The vertical grey line indicates the location of the scars.

7. QUANTIFYING FINITE-SIZE EFFECTS ON GRAIN-BOUNDARY SCAR CONFIGURATION, EDGE FLOW, AND BULK FLOW

To probe the impact of curvature of confinement on self-shear and scar configuration, we performed additional experiments for different system sizes: $N = 2000$, 1000, and 500 particles at $\phi = 0.72$ and $\chi = 1$. In all these experiments we observed azimuthally-aligned GB scars near the boundary at $r/R \approx 0.9$ (Supplementary Fig. 9A). Also, we observed self-shearing for $N = 2000$ particles, but not for the other system sizes (Supplementary Fig. 9B).

For $N = 1000$ and $N = 500$, we nonetheless see a sudden drop in $\omega(r)$ around $r/R = 0.9$; there is still a decoupling between the edge and the bulk mediated by GB scars. But for these smaller system sizes, the bulk rotates in the same direction as the edge, but with a smaller angular velocity.

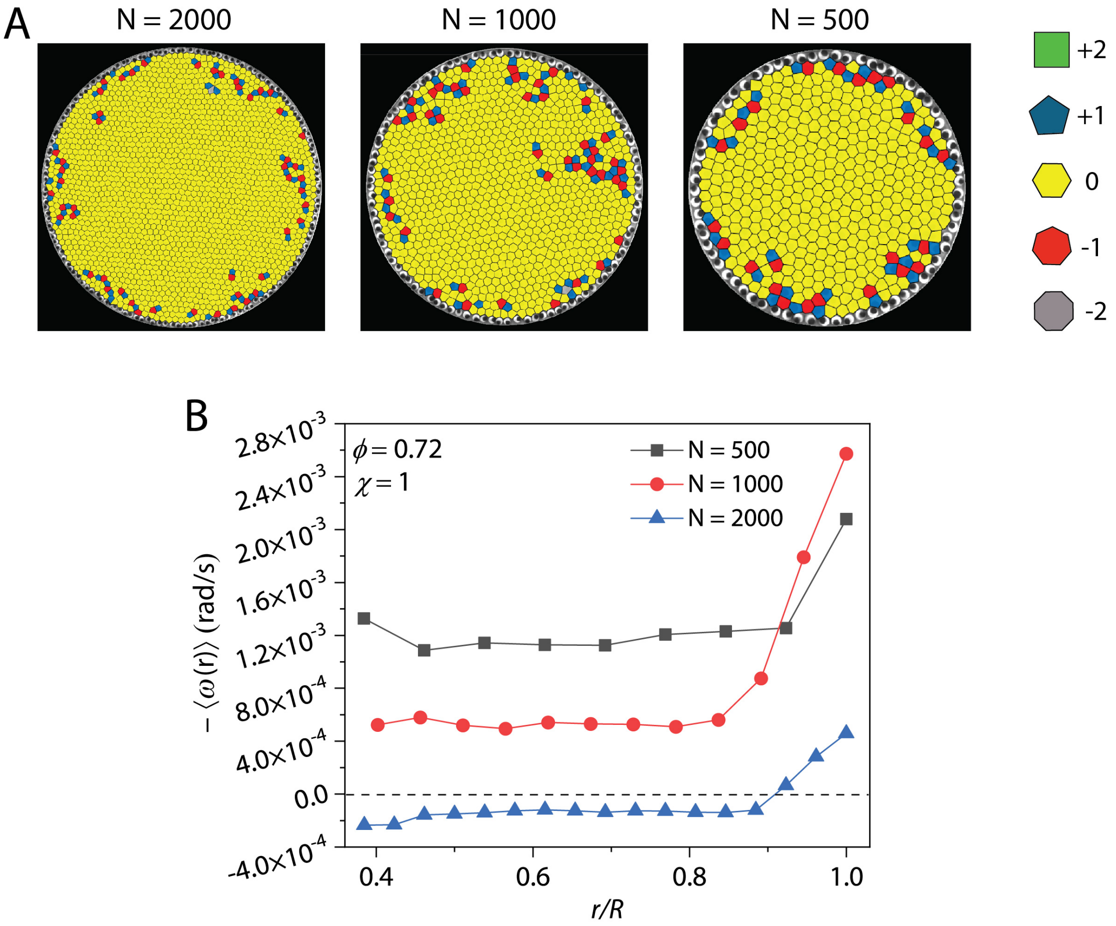

Supplementary Fig. 9: System size dependence of scar configuration for chiral spinners (experiment). (**A**) Voronoi tesselation of $\otimes$ spinners in the experiment at $\phi = 0.72$ for $N = 2000, 1000$, and 500. (**B**) The average angular velocity of spinners in concentric annuli with respect to the system center, $\omega(r)$, versus r/R at $\chi = 1$ and $\phi = 0.72$ for various system sizes, N.

8. PHASE DIAGRAM IN (χ, ϕ) PLANE

The phase diagram shows Voronoi tessellation of the spinners for χ ranging from 0 to 1 at $\phi = 0.68$, $\phi = 0.72$, $\phi = 0.75$, $\phi = 0.79$, respectively (Supplementary Fig. 10). The yellow hexagons represent six-coordinated spinners. The blue and red polygons are for five- and seven-coordinated spinners, representing disclination defects in a crystal. The total number of defects decreases monotonically with increasing ϕ at a constant χ. A reentrant was observed in the total number of defects when χ was increased for $\phi = 0.68$ and $\phi = 0.72$. The six-grain boundary scars became evident with an increase in ϕ.

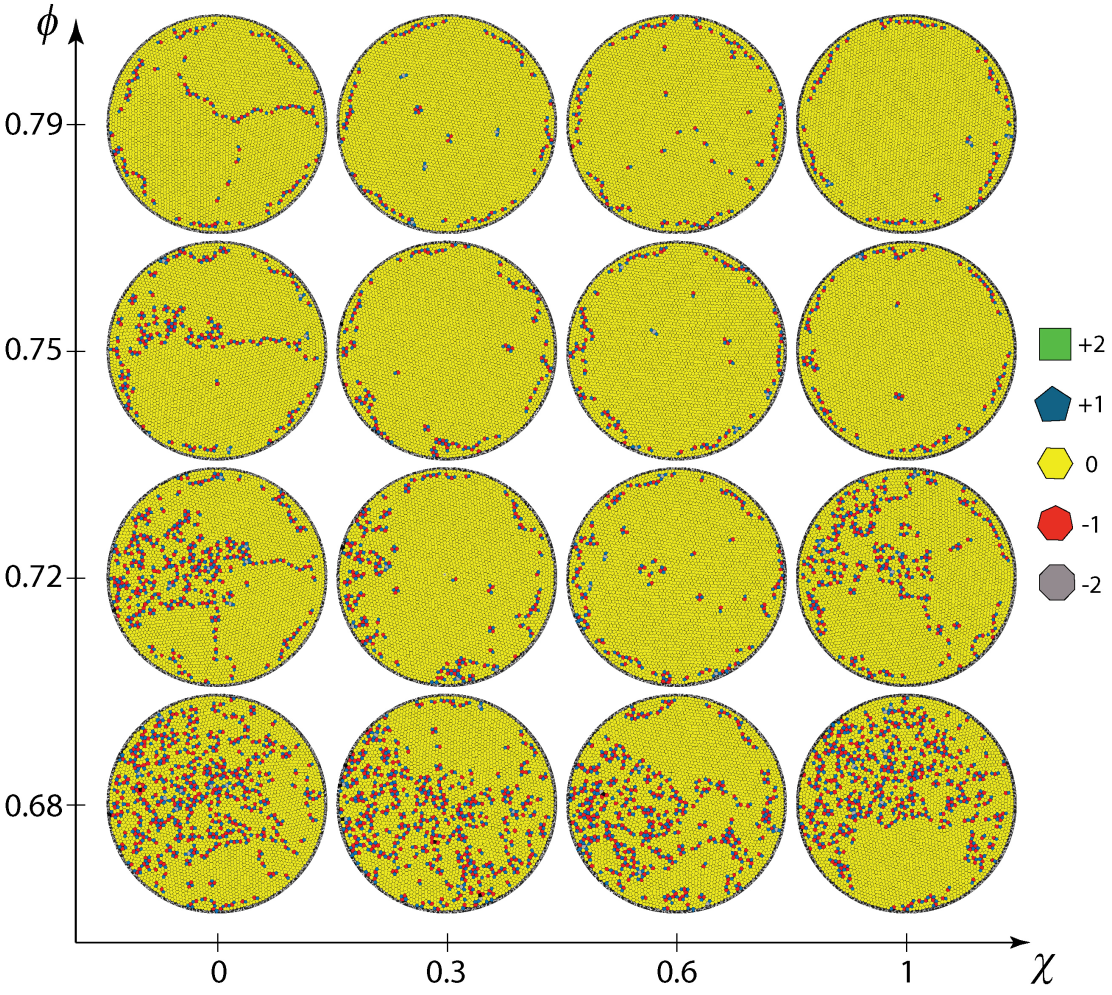

Supplementary Fig. 10: A reentrant melting at fixed ϕ as a function of χ. The phase diagram shows Voronoi tesselation of spinners at various χ for $\phi = 0.68$, $\phi = 0.72$, $\phi = 0.75$, and $\phi = 0.79$. The polygons are colored based on the coordination of spinners. The topological charge, $q_i = 6 - z_i$, where z_i is the coordination number of the spinner, is also indicated.

9. GRAIN BOUNDARY SCARS IN SIMULATIONS OF PASSIVE DISKS FOR DIFFERENT SYSTEM SIZES

We performed equilibrium simulations of passive disks under circular confinement at $\phi = 0.72$ for different extents of confinement (system sizes). Voronoi tessellations reveal azimuthally aligned scars near the boundary for $N = 2000$ and $N = 1000$, but less so for $N = 500$ (Supplementary Fig. 11). We also see defects in the bulk for $N = 2000$ across different runs. Importantly, like in the case of particle packings on a sphere (Ref. 9 and 14 of the main manuscript), the scar length decreases with increasing curvature.

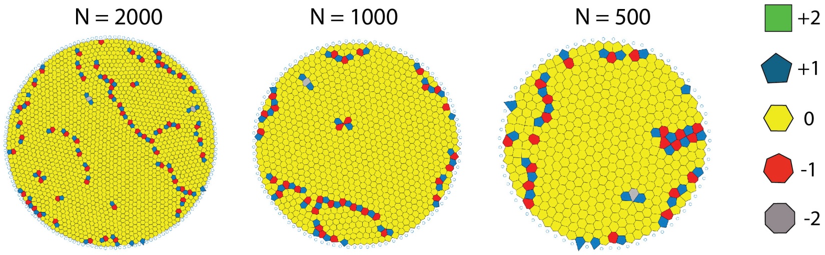

Supplementary Fig. 11: System size dependence of scar configuration for passive disks (simulations). Voronoi tesselation of simulated passive disks at $\phi = 0.72$ for $N = 2000, 1000$, and 500.

10. GRAIN BOUNDARY SCARS IN SIMULATIONS OF SPINNERS

In experiments, we observe azimuthally aligned grain boundaries or scars (string of dislocations). In our minimal simulation model, we investigate the robustness of the presence of such scars. To explore this, we performed simulations at $\phi = 0.72$ and $\phi = 0.79$, where the initial conditions were chosen from the corresponding experiments. In Supplementary Fig. 12 (Top panel), we have shown data for $\phi = 0.79$. We evolve the system for a long time $t = 2 \times 10^4$ (number of steps 10^7) at a high enough translational noise (for $D^T = 4 \times 10^{-3}$) to melt the initial crystalline structure completely (Top left panel of Supplementary Fig. 12). We then refreeze the system by quenching the translational diffusion constant $D^T = 2 \times 10^{-4}$ and evolve the system with reduced noise. Scars with similar features (number, length, location, orientation, etc.) then reappear, which demonstrates the robustness of the scars in a 2-D confined geometry. In Supplementary Fig. 12 (Bottom panel), we have shown a similar set of snapshots where the melting of the initial structure was done for $D^T = 10^{-3}$ and freezing was done at $D^T = 2 \times 10^{-5}$.

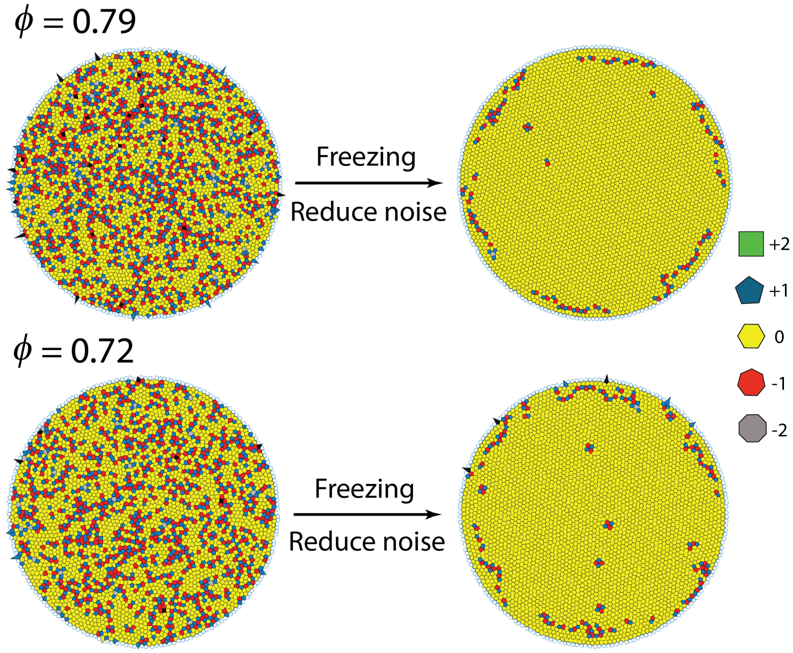

Supplementary Fig. 12: Observation of six scars in the simulation. Top panel: Voronoi tesselation before (left) and after (right) freezing at $\chi = 1$ and $\phi = 0.79$. Bottom panel: Voronoi tesselation before (left) and after (right) freezing at $\chi = 1$ and $\phi = 0.72$. The colors represent the spinner's coordination number. The topological charge for each spinner, $q_i = 6 - z_i$, where z_i is the coordination number of the spinner, is also indicated.

11. RADIAL DEFECT DENSITY

A simple way to quantify the azimuthally aligned grain boundary scars is to look at the radial defect density profile. Here, we count the total number of disclination defects (pentagons and heptagons in Voronoi tessellation) in concentric annuli to calculate the number density. Supplementary Fig. 13 shows the normalized annular defect density, $\rho_D(r)$, as a function of distance from the center, r/R. We observed a sharp peak in $\rho_D(r)$ at $r/R \approx 0.9$ (vertical gray line) for $\phi = 0.79$ corresponding to six-grain boundary scars present in the Voronoi diagram. These peaks are present at all the other values of ϕ and χ. As the defects are scattered over the entire system for lower values of ϕ, the peak height corresponding to $r/R \approx 0.9$ decreases with a decrease in ϕ. For $\phi = 0.68$, where the grain boundary scars are not evident, we still see a peak at $r/R \approx 0.9$.

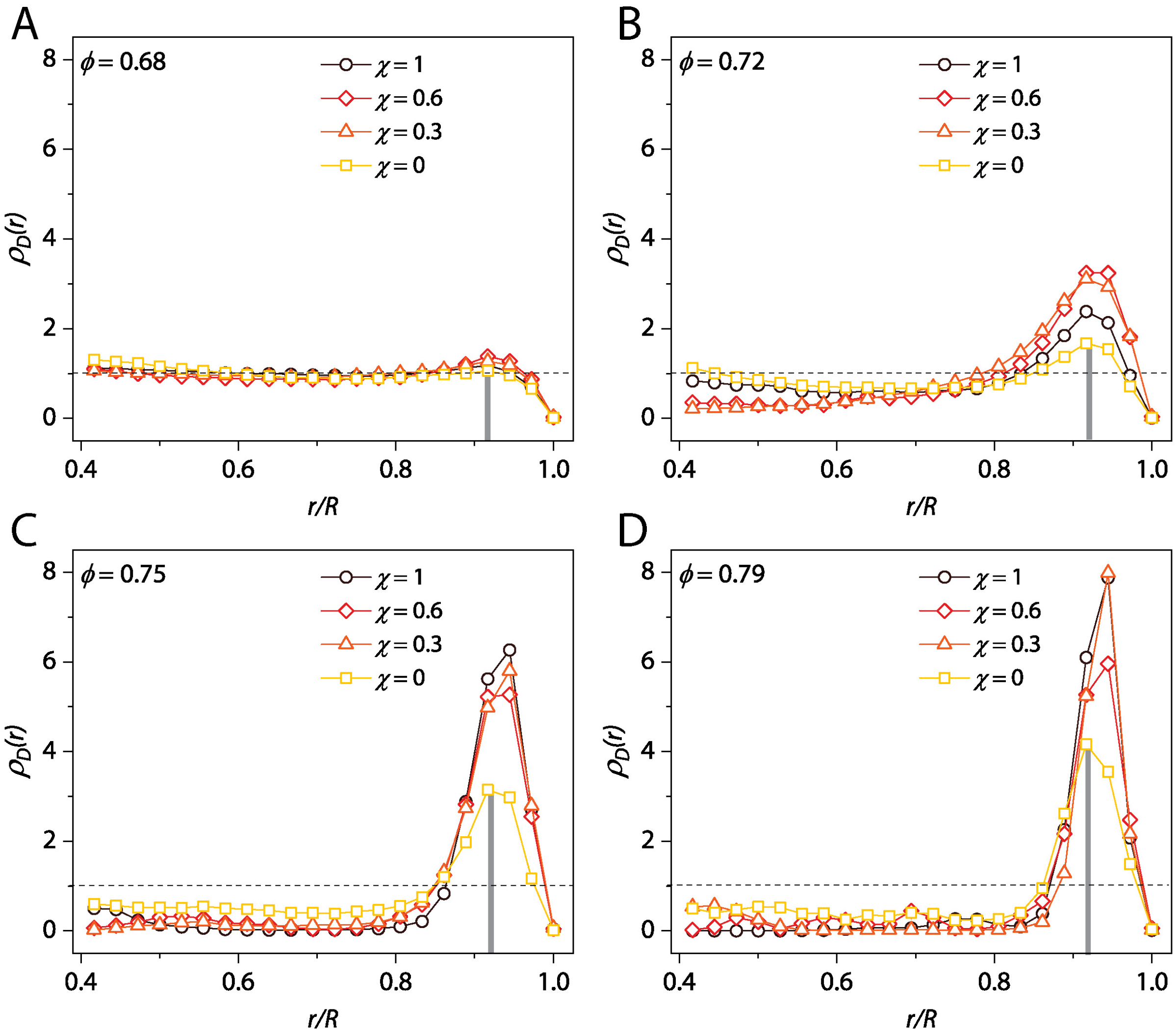

Supplementary Fig. 13: Grain boundary scars are consistent across all values of χ and ϕ. (**A** to **D**) Normalised defect density in concentric annuli, $\rho_D(r)$, versus r/R for various values of χ for (**A**) $\phi = 0.68$, (**B**) $\phi = 0.72$, (**C**) $\phi = 0.75$, and (**D**) $\phi = 0.79$. The number density in an annulus is averaged over all times.

12. RADIAL HEXAGONAL BOND-ORDER PARAMETER

We looked at the radial profile of the hexagonal bond-order parameter to gain insight into the radial crystallinity. Supplementary Fig. 14 shows the annular hexagonal bond-order parameter, $\langle|\psi_6^i|(r)\rangle$, calculated in concentric annuli as a function of distance from the center, r/R. The width of each annulus is roughly the same as the spinner diameter. We observed a sharp dip in $\langle|\psi_6^i|(r)\rangle$ at $r/R \approx 0.9$ (vertical gray line) for $\phi = 0.79$ corresponding to six-grain boundary scars present in the Voronoi diagram. Similar dips in the radial profiles of $\langle|\psi_6^i|(r)\rangle$ at all values of ϕ and χ suggest that grain boundary scars are robust to our experiments. We observed a χ-dependent radial profile for $\langle|\psi_6^i|(r)\rangle$ at all values of ϕ.

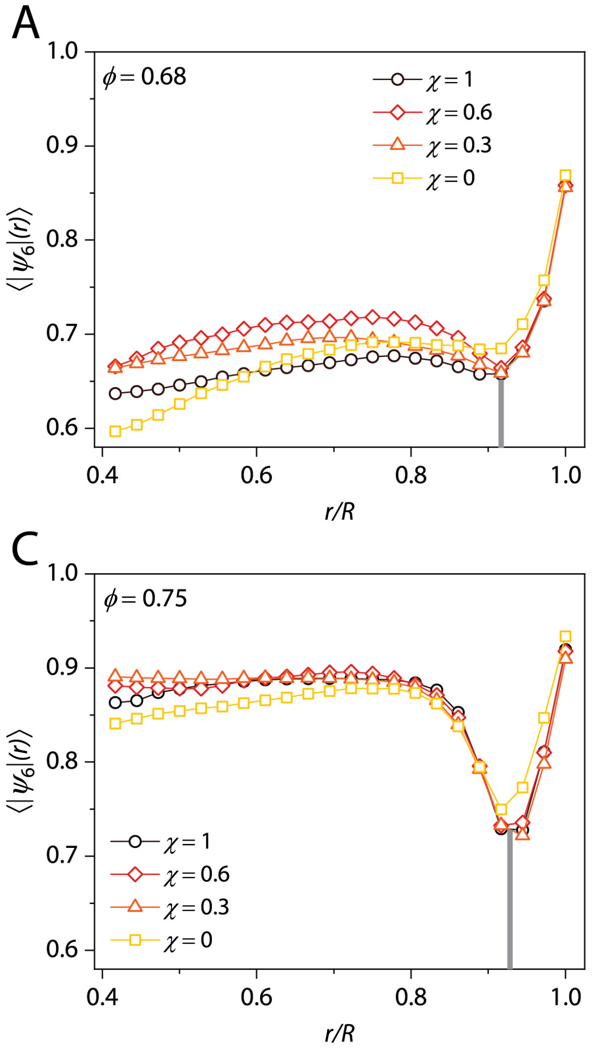

Supplementary Fig. 14: A χ-dependent radial crystallinity. (**A to D**) hexagonal bond-order parameter in concentric annuli, $\langle|\psi_6^i|(r)\rangle$, versus r/R for various values of χ for (**A**) $\phi = 0.68$, (**B**) $\phi = 0.72$, (**C**) $\phi = 0.75$, and (**D**) $\phi = 0.79$. Here, $\langle\rangle$ denotes an average over all the spinners in an annulus and at all times.

13. ODD MODULI IN SPINNER MATERIALS

In the continuum picture of solids and liquids, the stress tensor provides information about deformation and flow that lead to internal stress. The stress tensor, $\sigma_{ij} = K_{ijkl}\partial_l u_k + \eta_{ijkl}\partial_l \dot{u}_k + \cdots$, takes into account all the surface forces acting on a material element [5, 6]. Here, K_{ijkl} is the elasticity tensor that acts as a proportionality coefficient between the stress tensor and the displacement gradient, $\partial_l u_k$, while the viscosity tensor, η_{ijkl}, is the proportionality coefficient

between the stress and the velocity gradient, $\partial_l \dot{u}_k$. The viscosity and elasticity tensors in two dimensions contain $2^4 = 16$ independent components, respectively. Supplementary Fig. 15 shows a geometrical representation of stress that relates velocity gradient and displacement gradient through the viscosity and elasticity tensor in a 2D isotropic material that breaks parity. Here, stress σ_{ij} is represented by a vector that contains pressure ( ), torque density ( ), shear stress 1 ( ), shear stress 2 ( ). Similarly, displacement gradient $\partial_k u_l$ is represented by a vector that contains dilation ( ), rotation ( ), Shear strain 1 ( ), Shear strain 2 ( ). A $(\bullet)$ is placed above the shapes to represent the velocity gradient $\partial_l \dot{u}_k$ vector. The familiar coefficients in the typical fluid are shear, bulk, and rotational viscosities, η, ζ η_R, respectively, that form the diagonal elements of matrix η_{ijkl} (red symbols). The off-diagonal coefficients $\eta^1_\perp$ and $\eta^2_\perp$ couple rotation and compression (green symbols). The coefficient η_o couples the two independent shears. The off-diagonal coefficients $\eta^1_\perp$, $\eta^2_\perp$, and η_o all violate mirror symmetry. Similarly, the diagonal components of the matrix K_{ijkl} contain usual bulk, rotational, and shear moduli λ_0, μ_R, μ, respectively (red symbols). The antisymmetric contribution to the matrix K_{ijkl} comes from K_o and $K^2_\perp - K^1_\perp$. The two additional stresses are hydrostatic pressure p and the hydrostatic torque $2\eta_R\Omega$. Hydrostatic torque arises in fluids and solids made of spinning particles interacting through transverse forces.

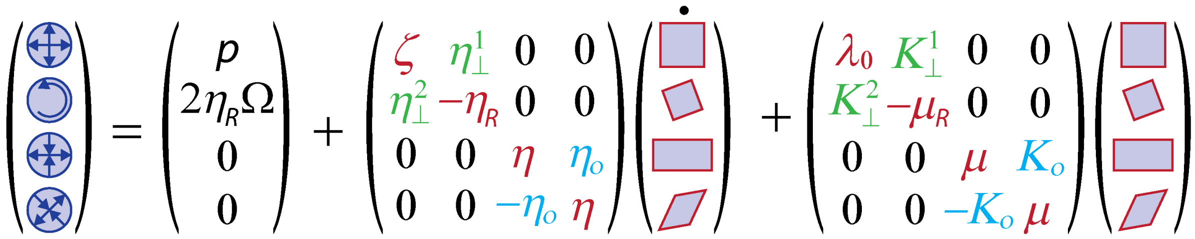

Supplementary Fig. 15: Velocity and displacement gradient relation with stress in a 2D isotropic material that breaks parity. Figure content adopted from [6].

14. GRAIN BOUNDARY SCARS AND SECTOR DENSITY

Grain boundary scars alter the radial density profile in our system. To quantify this effect, we divided our circular system into sectors, such that we have a sector that contains grain boundary scar (red shaded region in Supplementary Fig. 16A) and a sector that is free of scar (blue shaded region in Supplementary Fig. 16A). We then plotted the radial density, $\phi_A(r)$, in concentric annuli as a function of distance from the center, r/R, in these two sectors for $\phi = 0.68$ and 0.75 at $\chi = 1$. For both ϕ values, layering persisted into the bulk for the sector that did not have a scar (blue curves in the right panel of Supplementary Fig. 16A and B). For the sector that harbored a scar, radial density dropped after encountering a scar (red curves in the right panel of Supplementary Fig. 16A and B). The decrease in density reduces the coupling between layers near grain boundary scars, leading to a drop in η_R and $\sigma_{ij}^{\text{fric}}$.

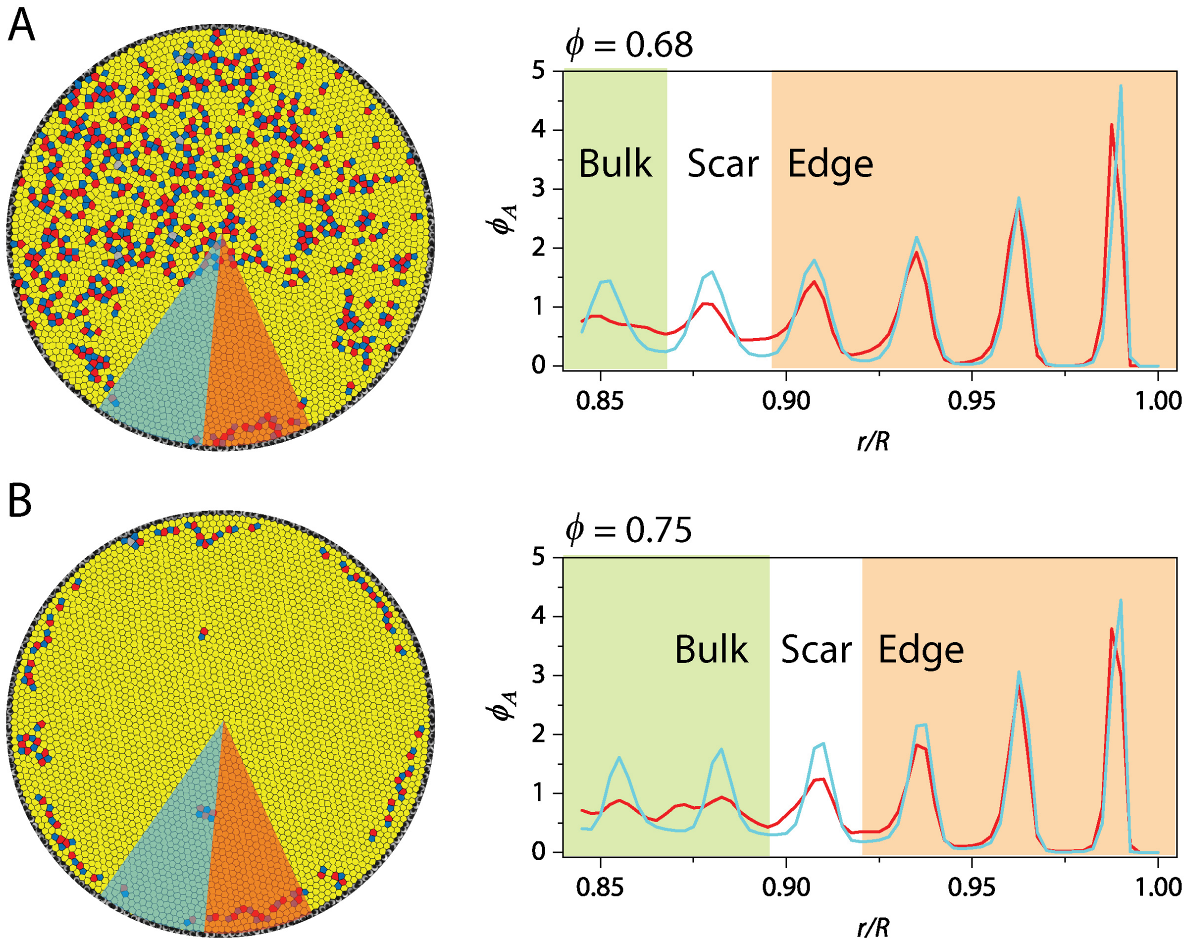

Supplementary Fig. 16: Grain boundary scars alters radial density profile. (**A and B**) Voronoi tesselation of spinners (left panel) and $\phi_A(r)$ verses r/R (right panel) at $\chi = 1$ for (**A**) $\phi = 0.68$, (**B**) $\phi = 0.75$. (**A and B**) The red-shaded region on the Voronoi tesselation represents the sector that contains a scar, while the blue-shaded region corresponds to a sector that is scar-free. The orange, white, and green shaded regions represent edge, scar, and bulk, respectively.

15. SIMULATIONS - RESISTIVE TORQUE PROFILE

The scars cause the edge flow to decouple from the bulk. To quantify the strength of coupling between layers, we analyzed the torque transfer between layers by measuring the pairwise torque arising from transverse frictional coupling. We computed the resistive torque experienced by a layer $\tau_{\text{res}}(r) = \sum_{i \neq j} \tau_{ij}$ where i denotes the index of

the spinners that belong to the annuli located at radius r and j indicates the index of spinners in the adjacent annuli. Supplementary Fig. 17 shows normalized resistive torque $\tau_{\mathrm{res}}(r)$ for $\chi = 1$ at various values of ϕ. We observed a clear drop in $\tau_{\mathrm{res}}(r)$ at $r/R \approx 0.9$ (gray-shaded region), indicating a decoupling between layers in the annuli containing GB scars.

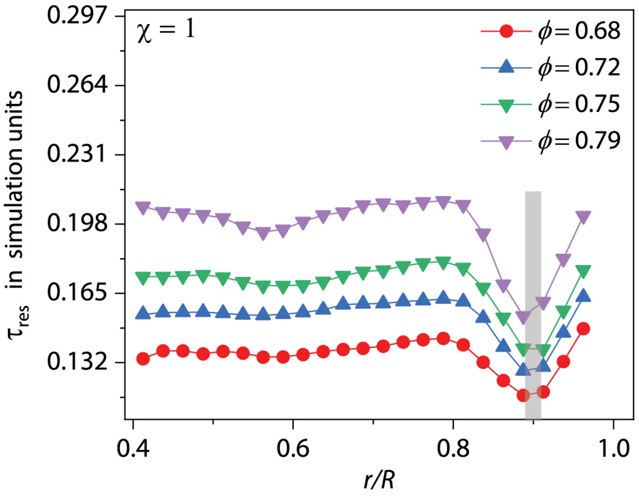

Supplementary Fig. 17: Scars weaken the coupling between layers. Resistive torque $\tau_{\mathrm{res}}(r)$ calculated as a function of radial distance r/R for $\chi = 1$ at $\phi = 0.68, 0.72, 0.75$ and 0.79. The grey-shaded region represents the annuli that contain the scars.

16. QUANTIFYING SPIN SPEEDS OF SPINNERS

A collision event between two ($\otimes$) spinners reduces the spin speeds of both spinners as the tangential forces from friction are in opposite directions (Supplementary Fig. 18A). In contrast, a collision event between a ($\otimes$) spinner and a ($\odot$) spinner enhances the spin speeds of both spinners. To quantify the χ dependence of angular speeds, we plot the probability distribution of spinner spin speed, $P(\Omega)$, for χ ranging from 0 to 1 at $\phi = 0.72$ (Supplementary Fig. 18B). The peak position (vertical dashed line) for $\chi = 0.3$ and 0.6 shows a maximum value of Ω, suggesting a reentrant in Ω as a function of χ. For $\chi = 1$, a $\otimes$ spinner is always surrounded by spinners of the same species, leading to a reduced spin speed (Supplementary Fig. 18A). For $\chi = 0.3$ and 0.6, a $\odot$ spinner will be surrounded mainly by $\otimes$ spinners, leading to an enhanced spin speed. For $\chi = 0$, the environment around a given spinner becomes homogeneous again, reducing the spin speed. We next plot the peak positions of the probability distribution of spinner spin speed $P(\Omega)$ at various values of ϕ as a function of χ (Supplementary Fig. 18C). At all the values of ϕ, the intermediate values of χ have maximum Ω, suggesting a reentrant in Ω as a function of χ. For a fixed value of χ, Ω decreases with an increase in ϕ.

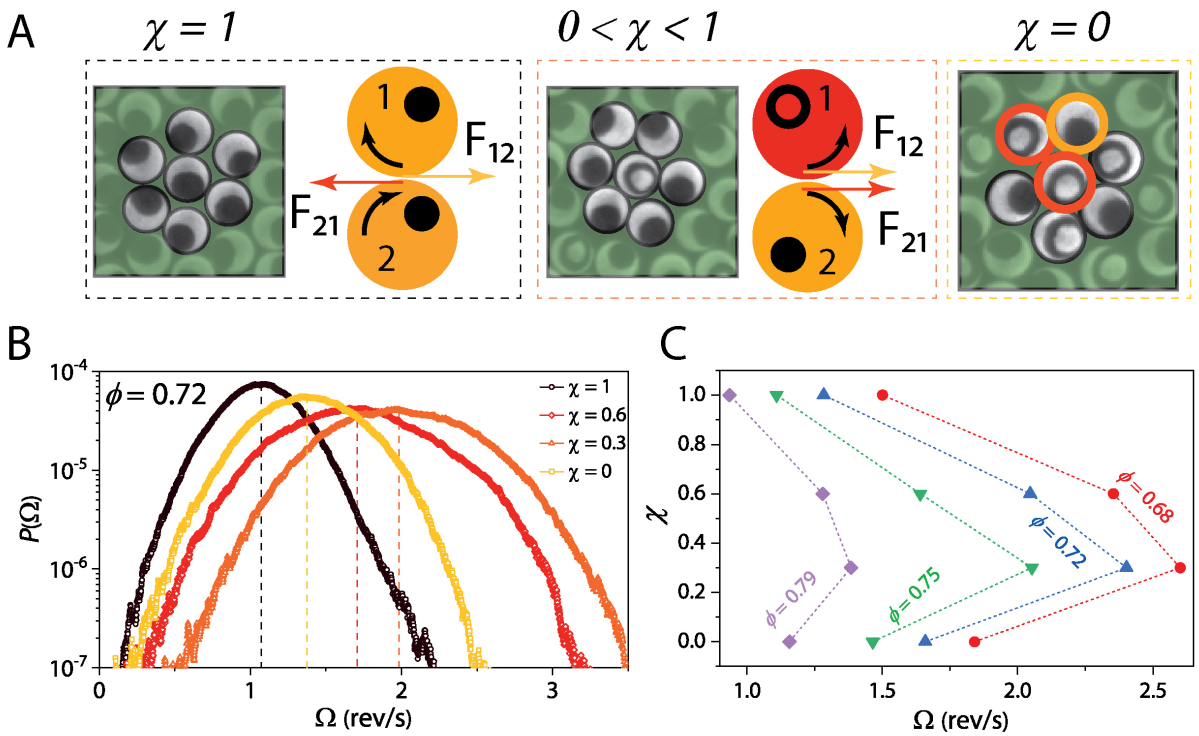

Supplementary Fig. 18: A reentrant in the spin speeds of spinners. (**A**) Local configuration of spinners for different values of χ along with the direction of tangential forces in a collision event between two spinners. (**B**) The probability distribution of spinner spin speeds $P(\Omega)$ at $\phi = 0.72$ for different values of χ. (**C**) χ versus Ω for various values of ϕ. The reentrant is present at all values of ϕ.

17. RADIAL PROFILE FOR SPIN VELOCITY OF SPINNER

The magnitude of the opposing tangential force between two layers depends on the average spin velocity of the spinners in a layer. To quantify this, we looked at the annular-averaged spinner spin velocity for χ ranging from 0 to 1 at $\phi = 0.68$, $\phi = 0.72$, $\phi = 0.75$, and $\phi = 0.79$ respectively (Supplementary Fig. 19). For all values of r/R, radial spinner spin velocity, $\langle\Omega(r)\rangle$, systematically decreases with χ. Although the spin speeds of the spinners are maximum for $\chi = 0.3$ (Supplementary Fig. 18), $\langle\Omega(r)\rangle$, is maximum for $\chi = 1$ as all the spinners are ($\otimes$). About 20%, 35%, and 50% of the spinners are ($\odot$) for $\chi = 0.6$, 0.3, and 0, respectively, which leads to the systematic decrease in $\langle\Omega(r)\rangle$ as a function of χ.

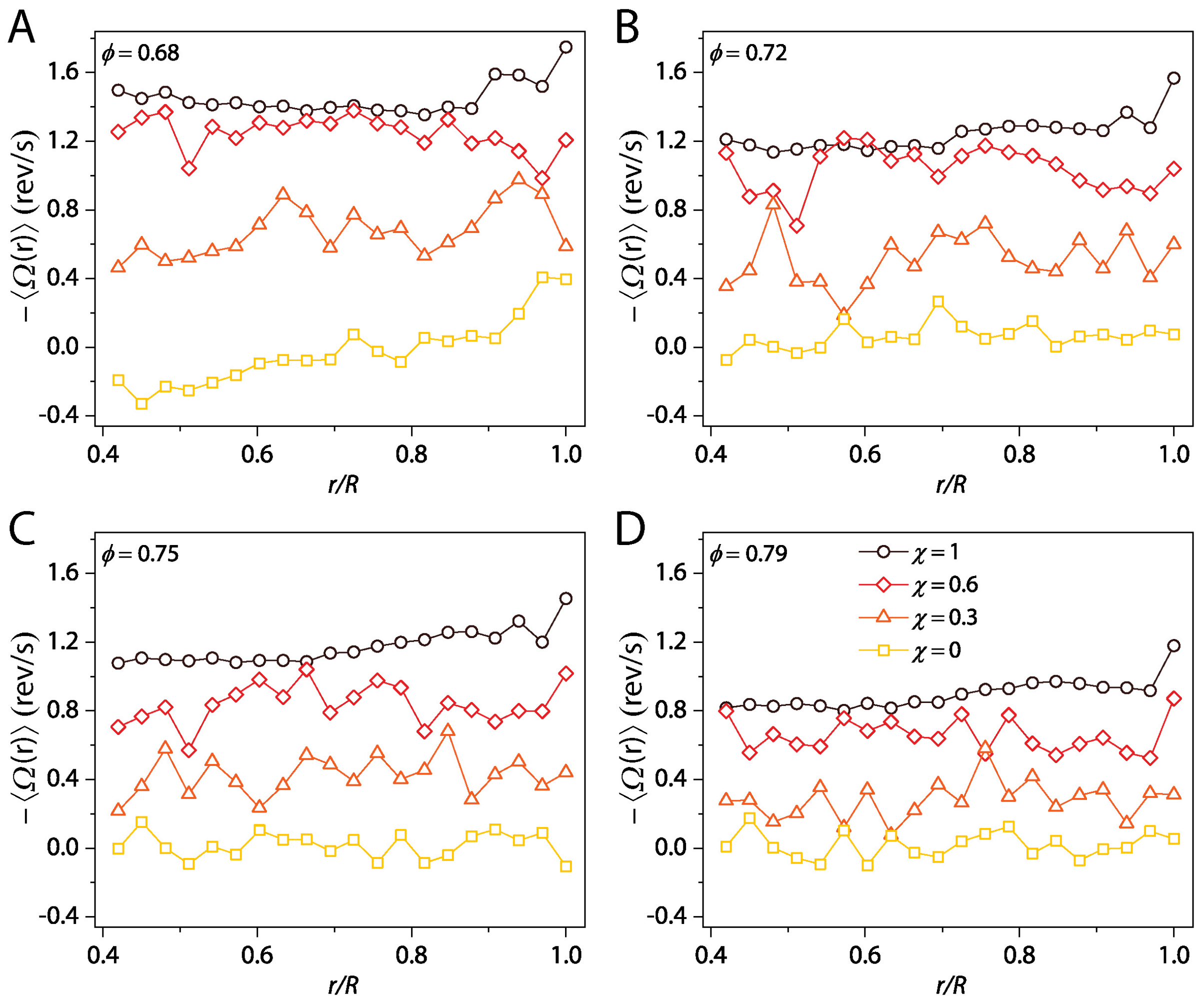

Supplementary Fig. 19: Annular spin velocity of spinners decreases with a decrease in χ. (**A to D**) The annular spin velocity of spinner $\langle\Omega(r)\rangle$ versus r/R for various values of χ for (**A**) $\phi = 0.68$, (**B**) $\phi = 0.72$, (**C**) $\phi = 0.75$, and (**D**) $\phi = 0.79$. Here, $\langle\rangle$ denotes an average over all the spinners in an annulus and at all times.

18. RELATING FLUCTUATIONS IN RADIAL SPIN AND ANGULAR VELOCITIES TO THE STRUCTURE OF THE SPINNER PACKING

We observed that both the annular angular and spin velocity profiles showed substantial fluctuations with r/R (see Fig. 2A and 2F). Focusing on the spin first, we note that the net spin velocity in a given annulus, $\langle\Omega(r)\rangle$, should depend on the total number of $\otimes$ and $\odot$ spinners, and hence, we expect it to correlate with the net chiral activity, $\chi(r)$, of that annulus. Supplementary Fig. 20A and B show annular spinner spin velocity and annular chiral activity as a function of distance from the center, r/R. For both $\chi = 0.3$ and 0.6, there is a one-to-one correspondence between the fluctuations in $\langle\Omega(r)\rangle$ and $\chi(r)$. Hence, these fluctuations have an underlying structural origin - the distribution of clockwise and counterclockwise spinners within each annulus.

For the annular angular velocity, we once again found a strong correspondence with the annular density fluctuations (Supplementary Fig. S20C, for $\chi = 1$ and $\phi = 0.72$). Here, however, this correspondence is more pronounced for $r/R > 0.6$. For $r/R \leq 0.6$, where there are many defects present, $\omega(r)$ shows large fluctuations (circles in Fig. 2A).

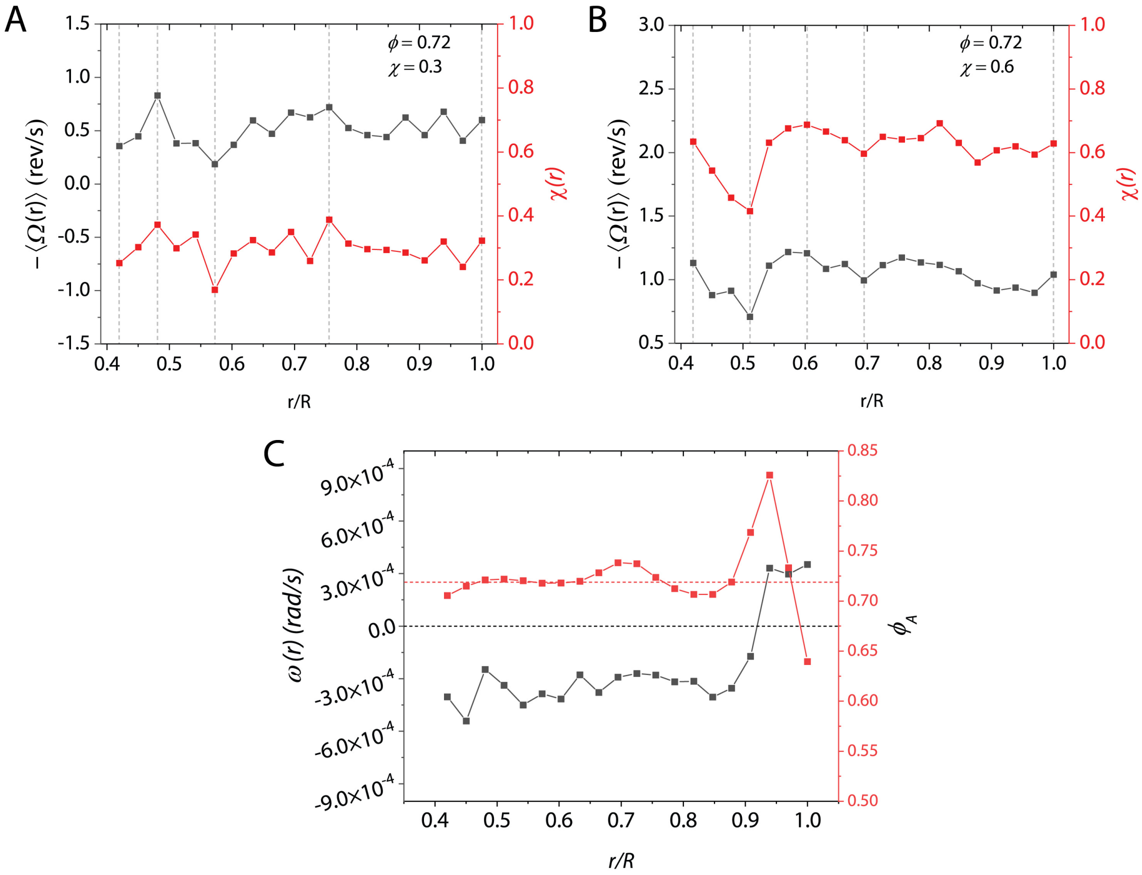

Supplementary Fig. 20: Fluctuations in $\langle\Omega(r)\rangle$ and $\omega(r)$ come from underlying fluctuations in $\chi(r)$ and $\phi_A(r)$, respectively. (**A to B**) Annular spinner spin velocity, $\langle\Omega(r)\rangle$, and annular chiral activity, $\chi(r)$, versus r/R at $\phi = 0.72$ for (**A**) $\chi = 0.3$, (**B**) $\chi = 0.6$. $\langle\Omega(r)\rangle$ profile is represented in black while the $\chi(r)$ profile is represented in red. (**C**) Annular angular velocity and annular area fraction versus r/R for $\chi = 1$ at $\phi = 0.72$. $\omega(r)$ profile is represented in black whereas the $\phi_A(r)$ profile is represented in red.

19. EXPERIMENTS - VORTICAL FLOWS AND RADIAL DENSITY PROFILES

Parity-violating collisions in chiral active systems can lead to an orthogonal response to the applied strain or stress [5–8]. Due to the presence of finite odd moduli (Supplementary Fig. 15), the emergent azimuthal flows can thus affect the radial density in our system. An azimuthal flow with the same handedness as the spinner spin produces a radially inward stress. In contrast, a mismatch between the handedness of azimuthal flow and the spinner spin causes radially outward stress (see Fig. 3A of the Main manuscript). To accurately represent the flows in our system, we compute vorticity from a coarse-grained velocity field. Since the longest-lived flows should mainly modify the radial densities, we calculate the vorticity corresponding to these longest-lived flows. The following section shows the correspondence between vorticity and radial density for $\phi = 0.79$, $\phi = 0.75$, and $\phi = 0.68$.

19.1. Vorticity and radial density at $\phi = 0.79$

Supplementary Fig. 21 (top panel) shows coarse-grained vorticity at $\phi = 0.79$ for various values of χ. The bottom panel of Supplementary Fig. 21 shows the peak height, P_H, of the radial density versus r/R for all χ values. To illustrate the behavior of P_H in bulk, we show the slope values obtained by fitting the P_H plot for $r/R < 0.8$. For $\chi = 1$, the edge flow extends to the bulk, and the system shows a rigid body-like rotation. This $\otimes$ flow leads to a radially inward stress $(-\sigma_{rr}\hat{r})$ and compresses the bulk, causing P_H to increase with decreasing r/R for $r/R < 0.8$. The negative slope at $\chi = 1$ reflects the enhanced bulk density. For $\chi = 0.6$, we observed similar $\otimes$ flow, but the

magnitude of flow is small. The bulk now is less compressed, and P_H is constant. For $\chi = 0.3$, we observed a rigid body-like rotation with handedness opposite to the earlier cases. The $\odot$ edge flow causes radially outward stress ($\sigma_{rr}\hat{r}$), making P_H to decrease with decreasing r/R for $r/R < 0.8$ - a clear signature of bulk dilation. For $\chi = 0$, the edge and bulk flow magnitude are zero as we have equal $\otimes$ and $\odot$ spinners. However, we observed small positive and negative vortices in different regions, but they did not impact the radial density. As expected, the slope value for P_H at $\chi = 0$ is between $\chi = 0.6$ and $\chi = 0.3$.

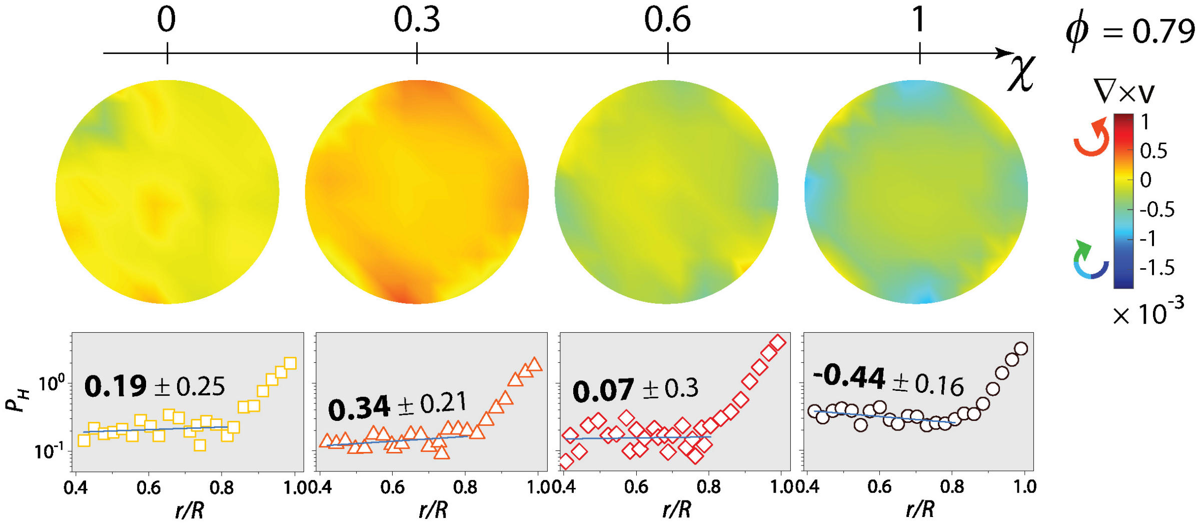

Supplementary Fig. 21: Odd stress modifies the radial density profile. Top panel: Vorticity field for various values of χ at $\phi = 0.79$. The color bar denotes the value of vorticity. Bottom panel: The peak height P_H of the radial density profile versus r/R at $\phi = 0.79$ for different values of χ. Blue lines are a linear fit to the P_H for $r/R < 0.8$. Arrows beside the color bar represent the handedness of the vortex.

19.2. Vorticity and radial density at $\phi = 0.75$

The top panel in Supplementary Fig. 22 shows the vorticity field, while the bottom panel shows the peak height, P_H, of the radial density versus r/R for values of χ ranging from 0 to 1 at $\phi = 0.75$. For $\chi = 0$, we observed small-sized positive and negative vortices spread across the system. The P_H was constant for $r/R < 0.7$, suggesting a flat density profile. For $\chi = 0.3$, $\otimes$ edge flow compresses the bulk, and the P_H increases with a decrease in r/R. For $\chi = 0.6$, we observed $\otimes$ edge flow but with a smaller magnitude than $\chi = 0.3$. The bulk is thus less compressed as compared to that of $\chi = 0.3$. We noticed one disparity at this stage. Even though there is a finite $\otimes$ edge flow at $\chi = 0.6$, we found that the value of the slope at $\chi = 0.6$ is greater than that at $\chi = 0$. To examine the extent of this disparity, we looked at the behavior of P_H in a different region of radial density (gray-shaded region in Supplementary Fig. 22B. The slope obtained from the linear fit of P_H is negative for $r/R < 0.6$ (inset plot in Supplementary Fig. 22B), implying that the discrepancy was only for a small window of r/R. To investigate further, we looked at the coarse-grained vortex flow (Supplementary Fig. 22D). The slightly asymmetric vortex flow about the system center could modify the radial density profile to bring a slight disparity. We observed a similar disparity even for $\chi = 1$ and again observed that the vortex flow was off-centered (Supplementary Fig. 22E).

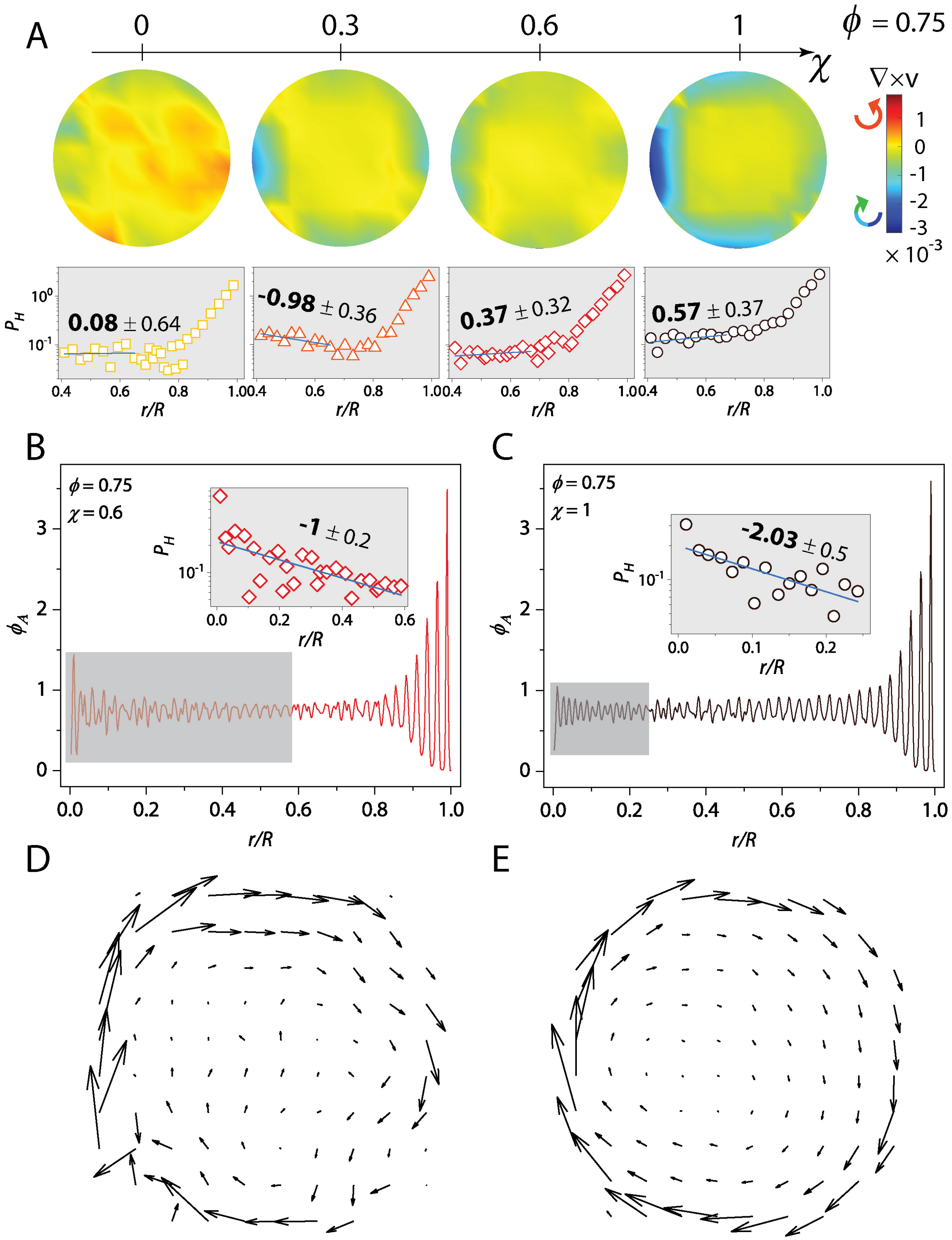

Supplementary Fig. 22: Odd stress alters the radial density profile. (**A**) Top panel: Vorticity obtained from a coarse-grained velocity field at $\phi = 0.75$ for various values of χ. The color bar denotes the value of vorticity. Bottom panel: The peak height P_H of the radial density profile versus r/R at $\phi = 0.75$ for different values of χ. (**B and C**) Radial density profile at $\phi = 0.75$ for (**B**) $\chi = 0.6$ and (**C**) $\chi = 1$. The inset plot shows the peak heights of the radial density profile, P_H, corresponding to the gray-shaded region. (**D and E**) Coarse-grained vortex flow at $\phi = 0.75$ for (**D**) $\chi = 0.6$ and (**E**) $\chi = 1$. The arrows represent the direction of the coarse-grained vorticity.

19.3. Vorticity and radial density at $\phi = 0.68$

In Supplementary Fig. 23(top panel), we plot vorticity from a coarse-grained velocity field at $\phi = 0.68$ for various values of χ. For $\chi = 0$, we observed weak positive and negative vortices in our system. The linear fit to P_H for $r/R < 0.7$ showed a positive slope. It is important to note here that the crystalline phase of the equilibrium hard disk in 2D is stable for $\phi^{\mathrm{Xtal}}_{\mathrm{Eq}} \geq 0.72$. Since $\phi = 0.68$ is well below $\phi^{\mathrm{Xtal}}_{\mathrm{Eq}}$, the bulk is mostly melted. As a result of layering near the wall, P_H decreases with decreasing r/R. For $\chi = 0.3$, we observed a $\otimes$ edge flow and a weak $\odot$ bulk flow. The P_H profile depends on the combined effect of inward and outward stress from the edge and the bulk flow. The linear fit to P_H for $r/R < 0.7$ again showed a positive slope. However, the value of the slope is smaller than the value observed for $\chi = 0$, suggesting that the $\otimes$ edge flow causes the bulk to compress. For $\chi = 0.6$, we observed a $\otimes$ edge flow and a weak $\odot$ bulk flow similar to $\chi = 0.3$. However, the $\otimes$ edge flow is lower in magnitude than that of $\chi = 0.3$. The bulk compression at $\chi = 0.6$ is less than $\chi = 0.3$, and the P_H decreases with a decrease in r/R for $r/R < 0.7$. For $\chi = 1$, we observed a $\otimes$ edge flow and a significant $\odot$ bulk flow. The edge flow leads to the inward radial stress, $-\sigma_{rr}\hat{r}$, that compresses the bulk. However, the large bulk flow creates an outward radial stress, $\sigma_{rr}\hat{r}$, that dilates the bulk. The slope value at $\chi = 1$ is thus greater than $\chi = 0.6$ and $\chi = 0.3$, indicating a reentrant melting transition at $\phi = 0.68$.

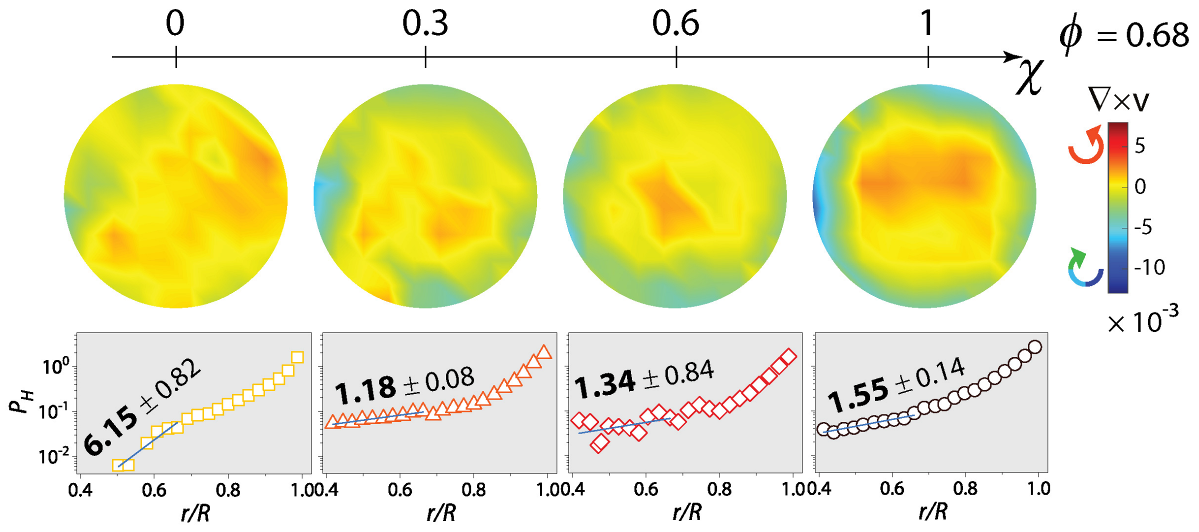

Supplementary Fig. 23: Odd stress drives the reentrant melting. Top panel: Vorticity obtained from a coarse-grained velocity field for various values of χ at $\phi = 0.68$. The color bar denotes the value of vorticity. Bottom panel: The peak height P_H of the radial density profile versus r/R at $\phi = 0.68$ for different values of χ.

20. SUPPLEMENTARY VIDEOS

The movies show the collective motion of the spinners in circular confinement from our experiment. The blue circles represent the spinners. We render a few spinners as filled circles for better flow visualization. The vertical red line indicates the initial location of the representative spinners. Movies are played at 75× real-time speed.

Supplementary Video 1 : The motion of the spinners at $\chi = 1$ and $\phi = 0.72$.
Supplementary Video 2 : The motion of the spinners at $\chi = 0.6$ and $\phi = 0.72$.
Supplementary Video 3 : The motion of the spinners at $\chi = 0.3$ and $\phi = 0.72$.
Supplementary Video 4 : The motion of the spinners at $\chi = 0$ and $\phi = 0.72$.

[1] K. Binder and W. Kob, *Glassy materials and disordered solids: An introduction to their statistical mechanics.* World scientific, 2011.
[2] J.-C. Tsai, F. Ye, J. Rodriguez, J. P. Gollub, and T. Lubensky, "A chiral granular gas," *Physical review letters*, vol. 94, no. 21, p. 214301, 2005.
[3] B. C. Van Zuiden, J. Paulose, W. T. Irvine, D. Bartolo, and V. Vitelli, "Spatiotemporal order and emergent edge currents in active spinner materials," *Proceedings of the national academy of sciences*, vol. 113, no. 46, pp. 12919–12924, 2016.
[4] K. Dasbiswas, K. K. Mandadapu, and S. Vaikuntanathan, "Topological localization in out-of-equilibrium dissipative systems," *Proceedings of the National Academy of Sciences*, vol. 115, no. 39, pp. E9031–E9040, 2018.
[5] M. Fruchart, C. Scheibner, and V. Vitelli, "Odd viscosity and odd elasticity," *Annual Review of Condensed Matter Physics*, vol. 14, no. 1, pp. 471–510, 2023.
[6] E. S. Bililign, F. Balboa Usabiaga, Y. A. Ganan, A. Poncet, V. Soni, S. Magkiriadou, M. J. Shelley, D. Bartolo, and W. T. Irvine, "Motile dislocations knead odd crystals into whorls," *Nature Physics*, vol. 18, no. 2, pp. 212–218, 2022.
[7] D. Banerjee, A. Souslov, A. G. Abanov, and V. Vitelli, "Odd viscosity in chiral active fluids," *Nature communications*, vol. 8, no. 1, p. 1573, 2017.
[8] C. Scheibner, A. Souslov, D. Banerjee, P. Surówka, W. T. Irvine, and V. Vitelli, "Odd elasticity," *Nature Physics*, vol. 16, no. 4, pp. 475–480, 2020.
