# Supplementary material for: Reentrant melting of scarred odd crystals by self-shear
Source: Nat Commun. 2026 Jan 16;17:1802. doi: 10.1038/s41467-026-68510-4 (PMC12916785; doi:10.1038/s41467-026-68510-4)
Supplement: Supplementary file 2 — Description of Additional Supplementary Files [file 41467_2026_68510_MOESM2_ESM.pdf]

Description of Additional Supplementary Files

Supplementary Video 1: The movies show the collective motion of the spinners in circular confinement from our experiment at $\chi = 1$ and $\phi = 0.72$. The blue circles represent the spinners. We render a few spinners as filled circles for better flow visualization. The vertical red line indicates the initial location of the representative spinners. Movies are played at 75× real-time speed.

Supplementary Video 2: The movies show the collective motion of the spinners in circular confinement from our experiment at $\chi = 0.6$ and $\phi = 0.72$. The blue circles represent the spinners. We render a few spinners as filled circles for better flow visualization. The vertical red line indicates the initial location of the representative spinners. Movies are played at 75× real-time speed.

Supplementary Video 3: The movies show the collective motion of the spinners in circular confinement from our experiment at $\chi = 0.3$ and $\phi = 0.72$. The blue circles represent the spinners. We render a few spinners as filled circles for better flow visualization. The vertical red line indicates the initial location of the representative spinners. Movies are played at 75× real-time speed.

Supplementary Video 4: The movies show the collective motion of the spinners in circular confinement from our experiment at $\chi = 0$ and $\phi = 0.72$. The blue circles represent the spinners. We render a few spinners as filled circles for better flow visualization. The vertical red line indicates the initial location of the representative spinners. Movies are played at 75× real-time speed.
